# An Improved Multispectral Palmprint Recognition System Using Autoencoder with Regularized Extreme Learning Machine

**DOI:** 10.1155/2018/8041609

**Published:** 2018-05-27

**Authors:** Abdu Gumaei, Rachid Sammouda, Abdul Malik S. Al-Salman, Ahmed Alsanad

**Affiliations:** ^1^Department of Computer Science, King Saud University, Riyadh, Saudi Arabia; ^2^Department of Information Systems, King Saud University, Riyadh, Saudi Arabia

## Abstract

Multispectral palmprint recognition system (MPRS) is an essential technology for effective human identification and verification tasks. To improve the accuracy and performance of MPRS, a novel approach based on autoencoder (AE) and regularized extreme learning machine (RELM) is proposed in this paper. The proposed approach is intended to make the recognition faster by reducing the number of palmprint features without degrading the accuracy of classifier. To achieve this objective, first, the region of interest (ROI) from palmprint images is extracted by David Zhang's method. Second, an efficient normalized Gist (NGist) descriptor is used for palmprint feature extraction. Then, the dimensionality of extracted features is reduced using optimized AE. Finally, the reduced features are fed to the RELM for classification. A comprehensive set of experiments are conducted on the benchmark MS-PolyU dataset. The results were significantly high compared to the state-of-the-art approaches, and the robustness and efficiency of the proposed approach are revealed.

## 1. Introduction

Biometrics is an effective technology used for security purposes in many applications [1]. Recently, it has gained more and more attention of researchers throughout the world. A number of biometric traits, including fingerprint, face, iris, gait, key-stroke, and palmprint, have been widely used according to the suitability of the applications [2–4]. Comparing to other biometric traits, palmprint has a strong stability, low distortion, and high uniqueness [5]. Unfortunately, palmprint patterns may be affected by some factors, such as variations in illumination, changes in orientation, and sensor noise that may lead to misclassification. Variations in illumination and changes in orientation of multispectral palmprint images can extremely affect the capability of such systems to recognize the individuals.

Several works have been proposed to solve these issues by using different feature extraction, reduction, and matching methods. These works can be categorized into four groups: line-based, statistic-based, subspace-based, and coding-based approaches.

The line-based approaches are proposed to detect the palmprint lines by using edge detector methods. For example, Han et al. [6] have proposed a method based on Sobel edge detector with morphological operations to extract the line features from palmprint images. Wu et al. [7] used the Sobel mask to compute the magnitudes of palmprint lines and project these magnitudes along both the *x*- and *y*-axes for generating the histograms as discriminative features.

Statistic-based approaches are also proposed in different studies and attained reasonable results [8–10]. There are several statistics that have been used in this group of approaches, such as Hu moments, Zernike moments, variance, mean, standard deviation, energy and histograms of local binary patterns. A number of transforms have also been utilized to extract the useful features from palmprint images. For example, Gan and Zhou [11] used a wavelet transform to convert the palmprint image into a small number of wavelet coefficients, then compute the variance and mean of these coefficients, and generate a feature vector of palmprint image. Li et al. [12] proposed a two-phase test sample representation (TPTSR) method as feature extractor for effective palmprint recognition. A Coarse to Fine *K*-Nearest Neighbor Classifier (CFKNNC) is proposed by Xu et al. [13] to improve the accuracy of palmprint recognition system. However, the CFKNNC is a more complicated than FKNNC because it consists of a large number of steps. Zhang and Gu [14] proposed a novel palmprint recognition method based on RBF kernel mapping function. In this method, a linear combination of all training dataset in the feature space is used to represent the testing dataset. A different set of methods based on texture features of palmprint is also introduced by some researchers in the area of palmprint recognition. These methods are proposed to compute the statistical features after extracting the palmprint textures using some filters and transforms such as Gabor wavelet, Gaussian derivative filters, wavelet transform, and Fourier transform. In this context, Xu et al. [15] proposed a method for palmprint recognition based on a quaternion matrix. This method utilized the principal components analysis (PCA) and wavelet transform for extracting the palmprint texture features from the quaternion matrix. For palmprint matching, the Euclidean distance classifier is used to compute the similarity between the extracted features.

Subspace-based methods are used to extract subspace features for improving the palmprint recognition system. Various representative subspace learning approaches such as principal components analysis (PCA), linear discriminant analysis (LDA), and independent component analysis (ICA). Lu et al. [16] proposed an approach to transform the original palmprint images into a small feature space set, called eigenpalms. In fact, the eigenpalms are the eigenvectors of the PCA, representing the training dataset of the palmprint images. In this method, Euclidean distance classifier is used for matching. In [17], a vertical and horizontal two-dimensional LDA (2DLDA) are applied to extract the Gabor features, then a distance-based adaptive method is employed to merge the vertical and horizontal features. Recently, Xu et al. [18] proposed a multispectral palmprint recognition approach using digital Shearlet transform and multiclass projection extreme learning machine. The approach used the singular value decomposition (SVD) to compute the projection matrix from the training dataset, after that, *k*th singular vector is extracted depending on the largest values in the singular matrix. Lu et al. [19] presented a multispectral palmprint recognition approach using fast and adaptive bidimensional empirical mode decomposition (FABEMD) method with tensor flow extreme learning machine (TFELM) classifier. In this approach, the multispectral images are decomposed into their bidimensional intrinsic mode functions by the FABEMD method; then the fusion coefficients are constructed at the decomposition level by using weighted Fisher criterion method. The experimental results of [18, 19] have demonstrated the capability of ELM classifier to recognize the palmprint patterns. However, ELM is not robust to translation, rotation, and other changes of palmprint templates and needs some regularization parameters for generality during the training phase. El-Tarhouni et al. [20] proposed a method for multispectral palmprint feature extraction. In this method, a kernel discriminant analysis (KDA) is used to reduce the dimensionality of features. For classification, a KNN classifier is used; however, it is not robust to inter- and intraclass variations of palmprint.

Coding-based approaches are the fourth group which is usually widely used in many works of palmprint recognition. Some coding methods are used to generate palmprint codes such as palm code [5], fusion code [21], ordinal code [22], competitive code [23], and Log-Gabor code [24]. For instance, Kong et al. [21] proposed a method for encoding the phases and responses of the six Gabor filters as a fusion code which is used later for competition. Jia et al. [25] proposed a robust line orientation code (RLOC) method to extract the orientation features of palmprint. A modified finite Radon transform is used in this method and the extracted feature vector is utilized as a competitive code. The classification step is performed using KNN classifier. However, the large size of features may lead to overfitting of palmprint classification. Hong et al. [26] proposed an approach to extract the palmprint orientation features. In this approach, rough feature and fine feature are extracted using Block Dominant Orientation Code (BDOC) and Block-based Histogram of Oriented Gradient (BHOG), respectively. Unfortunately, this approach may be affected by changes in illumination and shadowing of palmprint images. Fei et al. [27] proposed a half orientation code (HOC) for palmprint feature extraction. The authors used the half of Gabor filters to represent the HOC. Another method in [28] is introduced to extract the palmprint features based on a double orientation code (DOC) of Gabor filters with nonlinear classifier for matching. For evaluation, the methods in [27, 28] are tested using MS-PolyU database of multispectral palmprint images.

To improve the efficiency and performance of palmprint recognition system, we propose a novel multispectral palmprint recognition approach based on AE and RELM with efficient NGist descriptor. NGist is an extended version of Gist descriptor, used for describing the spatial envelope of the palmprint image. In NGist descriptor, we added a new step, named a variation tolerance step to cancel out the variation of average intensity values computed from different blocks of different scales and orientations. The variation tolerance step normalizes the palmprint features using Euclidean norm reducing the variations of features values due to changes in illumination, shadowing, and orientations. Hence, it can summarize the normalized features of scales and orientations for different parts of an image, providing a normalized rough description of the palmprint image. The NGist feature vector has a high dimension of features that increases the complexity of the classifier. To overcome this, we use the AE which not only help in dimensionality reduction but also address the nonlinearity of features. For recognition, a fast and robust regularized extreme learning machine (RELM) classifier is applied for palmprint recognition. In RELM classifier, a Frobenius norm is adopted as a regularization parameter to a trade-off between the approximated error and the regularized degree of the training samples.

The remainder of the paper is organized as follows: Section 2 explains the proposed multispectral palmprint recognition approach. The main steps of the approach based on NGist and AE with RELM are also introduced in this section.  Section 3 demonstrates the applicability of the proposed approach by a number of experimental models on a public database of multispectral palmprint images, named MS-PolyU. Finally, the conclusion and future work are summarized in Section 4.

## 2. Proposed Approach for Multispectral Palmprint Recognition 

**Figure 1:**
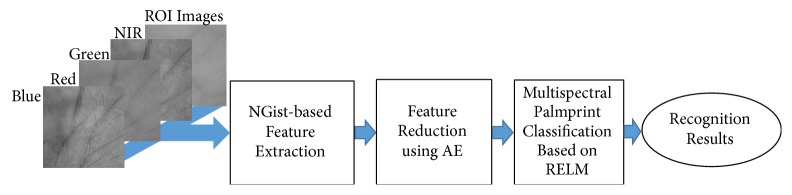
A diagram of the proposed approach for multispectral palmprint recognition.

The improved palmprint recognition system is based on the optimal spectral band which attains the highest recognition rate. The proposed approach starts after segmenting the region of interests (ROIs) from all spectral bands images by using Zhang et al.'s method [5]. The main steps of the proposed approach can be labeled as follows: NGist-based feature extraction, feature reduction using AE, and multispectral palmprint classification based on RELM.  Figure 1 shows the diagram of the proposed approach and its fundamental steps.

In NGist-based feature extraction, we extract the palm features by using effective NGist descriptor. Then, an optimized AE method is adopted to reduce the size of the extracted NGist feature vector (i.e., dimensionality of features).

Finally, in the classification step, the correct recognition of a person is accomplished when its palmprint image matches a palmprint image of the same person in the training dataset. While the incorrect recognition of a person may happen when its palmprint image does not match any palmprint image either for the same person or not or matches the palmprint image of another person in the training dataset. The classification step of the proposed approach is based on the RELM classifier. The advantages of RELM such as the speed for training and testing and the generality achieved by the regularization are exploited for improving the palmprint recognition system.

### 2.1. NGist-Based Feature Extraction

Global image features can be summarized by characterizing several substantial statistics of the input image. One of the methods used for feature extraction is the convolution process of the Gabor filter with an image at different scales and orientations. Consequently, the high and low frequency responses of gradient directions are measured as discriminative features. Taking the average intensity values of the convolution filter at each scale and orientation generates the Gist feature vector of an image. This feature vector is commonly being utilized for the image classification [29–31]. The first step of the proposed feature extraction method is converting the input image into a grayscale image. Then, the grayscale image is processed by a whitening filter and normalized according to the local contrast for preserving the main structural details. After that, it passed through a number of 2D Gabor filters of *m* scales with *n* orientations. The 2D Gabor filter function, *g*(*x*, *y*), and its Fourier function transform, *G*(*u*, *v*), can be calculated using (1) and (2), respectively:

(1) gx,y=12πσxσyexp⁡−12x2σx2+x2σy2+2πjWx,

where $j=\sqrt{-1}$ and *W* is the radial frequency of the Gabor function.

(2) $$\begin{array}{c} G\left(\;u,v\;\right)=\exp\left\{\;-\frac{1}{2}\left[\;{\frac{\left(u-W\right)}{\sigma_{u}^{2}}}^{2}+\frac{v^{2}}{\sigma_{v}^{2}}\;\right]\;\right\}, \end{array}$$

where *σ*_*u*_ = (1/2)*πσ*_*x*_, *σ*_*v*_ = (1/2)*πσ*_*y*_; with the Gabor functions, a complete and nonorthogonal basis set can be formed. Using this basis set, the signals can be expanded to provide with the best description of the localized frequency. Consider *g*(*x*, *y*) as a mother Gabor wavelet. Subsequently, self-similar filter dictionary can be obtained by suitable rotation and dilation parameters (orientation *θ* and scaling factor *α*) of following function:

(3) $$\begin{array}{c} g_{mn}\left(\;x,y\;\right)=\alpha^{-m}g\left(\;x^{\prime },y^{\prime }\;\right), \end{array}$$

where *α* > 1, *m*, *n* are integers and *x*′ and *y*′ are given by (4) as

(4) x′=α−mxcos⁡θ+ysin⁡θ,y′=α−mxsin⁡θ+ycos⁡θ,

where *θ* = *nπ*/*K* and *K* represents the number of orientations. The scale *α*^−*m*^ in (3) is aimed to guarantee that the energy is independent of *m*. The Gabor wavelets nonorthogonality indicates that there is a small redundancy among information of the filtered images, and the following strategy can be applied to reduce this redundancy of information. Consider the lower and upper center interest frequencies are indicated by *U*_*l*_ and *U*_*h*_. *K* and *S* represent the number of orientations and the number of scales in the multiresolution decomposition, respectively. Using (5), the projection of filters in the design strategy can be used to ensure that filter responses in the half-peak magnitude of the frequency spectrum touch one another.

(5) α=UhUl1/S−1,σu=α−1Uhα+12ln⁡2,σv=tan⁡π2KUK−2ln⁡2σu2Uh·2ln⁡2−2ln⁡22σu2Uh2−1/2,

where *W* = *U*_*h*_ and *m* = 0, 1, …, *S* − 1. For multispectral palmprint feature extraction, Gabor filters with different scales and orientations can be used for ensuring maximum information with minimum redundancy. The proposed method uses 4 scales (*m* = 0,1, 2,3) and 8 orientations (*n* = 0,1,…, 7) of the Gabor wavelet, resulting in a total of 32 Gabor images for each input image. These generated images are divided into a 4-by-4 block. For each block, the average intensity value is calculated to represent the feature of that block. The final output is a concatenated features vector, named Gist of 32 × 4 × 4 = 512 dimensions.

To cancel out the variation of average intensity values for blocks computed from different scales and orientations, we extend the traditional Gist descriptor by adding a new step, called variation tolerance step. In this step, the Gist features are normalized by using Euclidean norm as in Eq. (6). This extended version of Gist descriptor is named a normalized Gist (NGist) descriptor.

(6) $$\begin{array}{c} NGist=\frac{Gist}{\sqrt{\sum_{i=1}^{512}{\left|Gist\left(i\right)\right|}^{2}}} \end{array}$$

It is worth mentioning that we chose the Euclidean norm because it is the natural norm associated with the dot-product that measures the similarity between objects.

### 2.2. Feature Reduction Using AE

**Figure 2:**
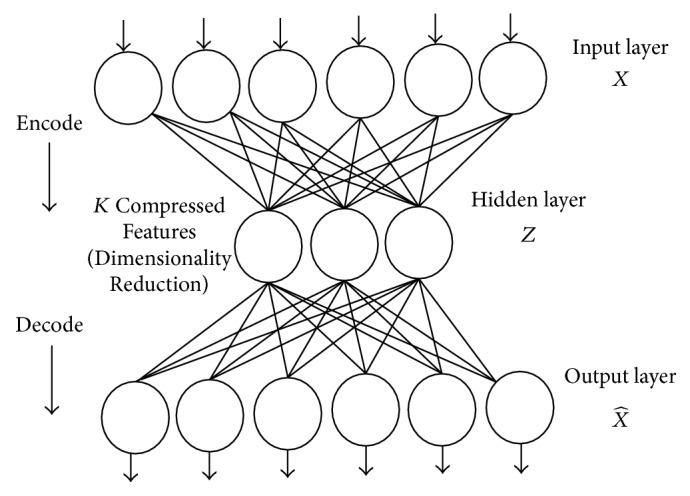
A simple autoencoder consists of a single hidden layer, input layer, and output layer.

AE is a feedforward neural network (FNN), utilized for unsupervised learning as an efficient encoding algorithm [32, 33]. The goal of AE is to learn the feature representation, especially for feature dimensionality reduction.  Figure 2 shows a simple form of AE consists of a single hidden layer, input layer, and output layer and.

In this subsection, a nonlinear AE is practically utilized to reduce the dimensionality of the NGist feature vector. New features from the extracted features of training and testing data samples are produced, separately. There are several parameters for AE should be tuned and prepared through this step. The key parameters of AE include the activation function, the number of hidden nodes, the weight decay and regularization parameter, the weights of hidden nodes, the amount of epochs to be iterated, and the learning rate.

Now, suppose that the structure of the AE as shown in Figure 1, the input vector, *X* ∈ *ℛ*^512^, represents the extracted NGist feature vector, and the output, $\hat{X}\in {ℛ}^{512}$, represents the reconstructed feature vector. Because we have only *K* hidden nodes, the AE is subjected to learn a compressed feature vector, *Z* ∈ *ℛ*^*K*^ (new feature vector), to recover 512-features of input, *X*. This new feature vector will be the input to the RELM for the classification task.

### 2.3. Multispectral Palmprint Classification Based on RELM

RELM is a feedforward neural network (FNN) consists of a single hidden layer, input layer and output layer. The weights of the input layer will be initialized randomly and the weights of the output layer can be computed arithmetically [34].

Suppose that *X* ∈ *ℛ*^*N*×*K*^ is a matrix of training dataset. The RELM with *K* hidden nodes and activation function, *g*(*x*), can be modeled by the following equation:

(7) $$\begin{array}{c} o_{j}=\overset{K}{\underset{i=1}{\sum }}\beta_{i}g\left(\;w_{i}\cdot x_{j}+b_{i}\;\right),\;j=1,\ldots ,N, \end{array}$$

where *w*_*i*_ = [*w*_*i*1_, *w*_*i*2_,…,*w*_*iK*_]^*T*^is a weight vector which connects each hidden node *i* with all input nodes, *w*_*i*_ · *x*_*j*_ represents an inner product of *w*_*i*_ and *x*_*j*_, *b*_*i*_ is a threshold of the *i*th hidden nodes, and *β*_*i*_=[*β*_*i*1_, *β*_*i*2_,…,*β*_*iN*_]^*T*^ is a weight vector between the *i*th hidden nodes and the output nodes. The *N* data samples can be approximated with zero error by

(8) $$\begin{array}{c} \overset{N}{\underset{j=1}{\sum }}\left\|\;o_{j}-t_{j}\;\right\|=0, \end{array}$$

where *t*_*j*_ is an encoding of the user's ID as a target vector. In order to represent this encoding uniformly, we define the target vector corresponding to users' IDs (id_*j*_) as

(9) $$\begin{array}{c} t_{j}={\left(\;b_{1},\ldots ,b_{i},\ldots ,b_{m}\;\right)}^{T}, \end{array}$$

where *m* is a number of users in the training set and *b*_*i*_ is equal to 1 or −1 depending on whether the related user's ID belongs to the corresponding IDs or not.

For example, suppose that there are three users (*m* = 3); the users' IDs, *t*_*j*∈(1,2,3)_ = (1,2, 3), and the related target vectors are as follows: *t*_1_ = (1, −1, −1)^*T*^ belongs to the first user, *t*_2_ = (−1,1, −1)^*T*^ belongs to the second user, and *t*_3_ = (−1, −1,1)^*T*^ belongs to the third user.

Equation (7) can be reformulated shortly as matrices by

(10) $$\begin{array}{c} T=H\beta , \end{array}$$

where

(11) H=⋯gwK·x1+bK⋮⋯⋮gw1·xN+b1⋯gwK·xN+bKN×K

(12) β=⋮βKTK×N,

where *H* is an output matrix of the hidden layer where each *i* column of *H* represents the output of the *i* hidden node related to the inputs, *x*_1_, *x*_2_,…, *x*_*N*_. The parameters of hidden nodes with nonzero activation functions, *g*(·), can be fixed randomly and the output weights can be computed on any input data sets [34, 35].

For testing phase, the output target matrix can be computed by

(13) $$\begin{array}{c} Y=\hat{H}\hat{\beta }\text{,} \end{array}$$

where $\hat{H}$ is an output matrix of the hidden layer for the testing dataset and $\hat{\beta }$ is a matrix of weights for the testing dataset which is computed as

(14) $$\begin{array}{c} \hat{\beta }={\left(\;H^{T}H+\lambda I\;\right)}^{-1}H^{T}T, \end{array}$$

where *I* is an identity matrix, *H*^*T*^ is a transpose matrix of *H*, *T* is a target output matrix, and *λ* is a regularizer parameter to trade-off between the regularized degree and the estimated error [36]. Frobenius norm is adopted as a regularization method in our work. This is because its efficiency to deal with sparse weights values.

For encoding the users' IDs (multilabel), we define each user's ID by using a discriminative target function written as

(15) $$\begin{array}{c} t_{j}=\underset{i}{\arg}\max\left(\;Y\;\right). \end{array}$$

Algorithm 1 describes the phases and steps of the classification task of the proposed approach.

## 3. Experimental Models and Discussion

A complete set of experimental models are done on the public database of multispectral palmprint, created by Hong Kong Polytechnic University (MS-PolyU) [5]. To evaluate and validate the robustness and efficiency of the proposed approach, we systematically establish three different experimental models during our experiments.

### 3.1. MS-PolyU Database Description

**Figure 3:**
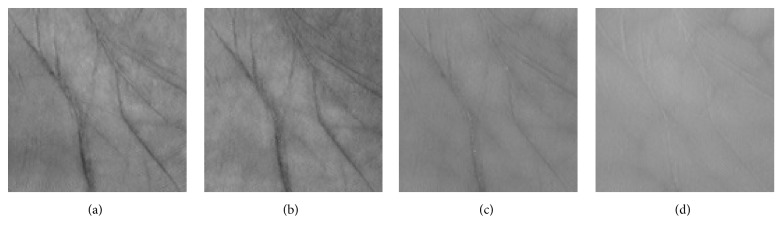
ROI images of a palm sample from the MS-PloyU database: (a) blue, (b) green, (c) red, and (d) NIR.

The database consists of 24000 multispectral images of 250 people, each with two different palms, taken under four varied illuminations: red, green, blue, and NIR spectral bands. The images were acquired from 55 females and 195 males in two separated sessions. In every session, six images were collected for each spectrum of each palm. The average interval time between the two sessions was about nine days. So, there is in total 250 (volunteers) × 2 (different palms) × 4 (different spectra) × 6 (images) × 2 (sessions) = 24,000 images within the MS-PolyU database.  Figure 3 shows the ROI images of a palm sample taken from the MS-PloyU database.

### 3.2. Parameters Setup

The number of hidden nodes in AE, *K*_AE_, is fixed to 250, since it is enough to represent the most important extracted features of palmprint. Another most important parameter is the number of hidden nodes within the RELM, *K*_RELM_. This parameter is selected using a grid search technique depending on the maximum accuracy. The grid search technique is done by using 71 different numbers of hidden nodes, ranging from 800 to 1500 with a common nonlinear sigmoid activation function.  Table 1 states all parameters which are initialized in our experiments.

### 3.3. Experimental Model I

In this experimental model, six samples images of each palm from the first session are taken as a training dataset, while the six samples images of each palm from the second session are taken as a testing dataset. So, there is in total 3000 (500 × 6) training images and 3000 (500 × 6) testing images for each spectral band (red, green, blue, and NIR illuminations). The results of recent previous works, such as TPTSR [12], NFS [13], DWT [15], FABEMD + TELM [19], LBP-HF + Gabor [20], Log-Gabor + *D*_Hamm_ [24], and Log-Gabor + *D*_KL_ [24] are used to compare and evaluate the results of the proposed approach according to this model.

**Figure 4:**
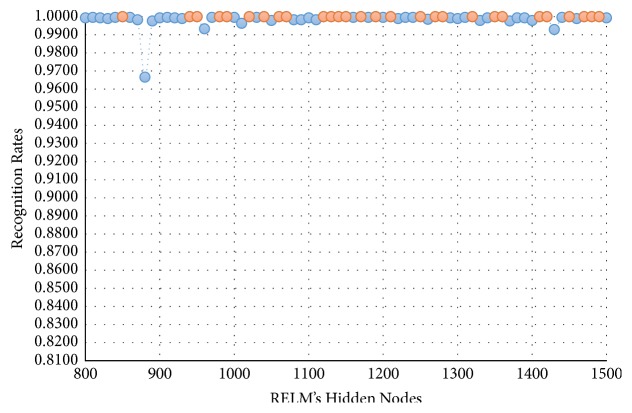
Recognition rates of blue spectral band using different numbers of RELM's hidden nodes.

**Figure 5:**
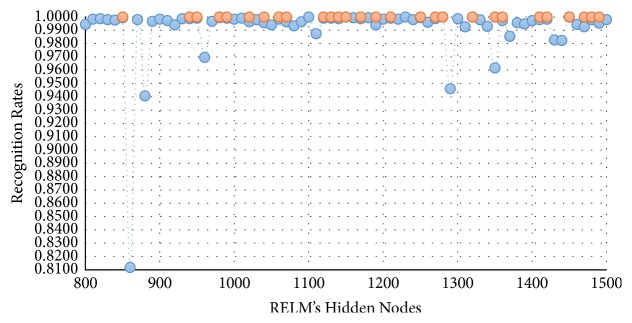
Recognition rates of green spectral band using different numbers of RELM's hidden nodes.

**Figure 6:**
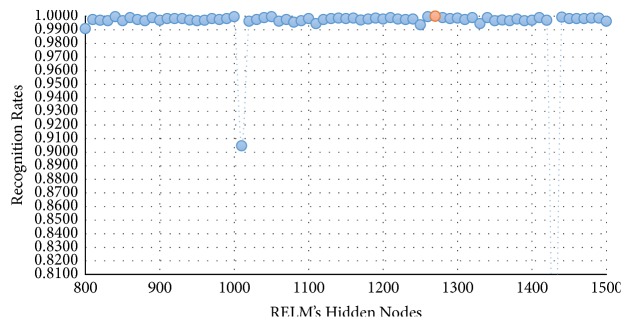
Recognition rates of red spectral band using different numbers of RELM's hidden nodes.

**Figure 7:**
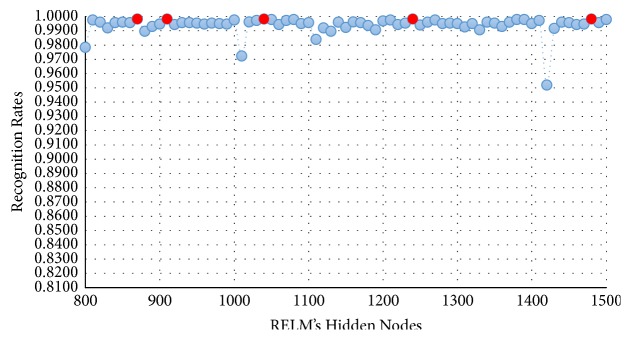
Recognition rates of NIR spectral band using different numbers of RELM's hidden nodes.

In this experimental model, we study the influence of the number of hidden nodes on the recognition accuracy. As we see in Figures 4–7, the highest recognition rates are highlighted and marked as orange and red circles. Orange circles mean that the highest accuracy value is equal to the maximum accuracy (100%). In Figures 4 and 5, there are a large number of high accuracy values, reaching the maximum value for both blue and green spectral bands. For red spectral band, there is only one maximum recognition rate when the number of hidden nodes is 1270. Nevertheless, the recognition rate, 99.97%, is also obtained with different numbers of hidden nodes. For NIR spectral band in Figure 7, the highest accuracy value is 99.83% which is repeated five times with five different numbers of hidden nodes (870, 910, 1040, 1240, and 1480). Although this accuracy value did not attain the maximum accuracy (100%), most of recognition rates are higher than 99.50%.

**Figure 8:**
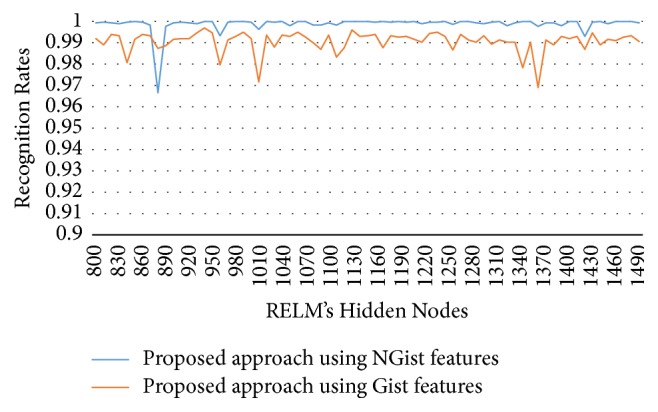
Recognition rates of blue spectral band using extracted features of NGist and Gist at different numbers of RELM's hidden nodes.

**Figure 9:**
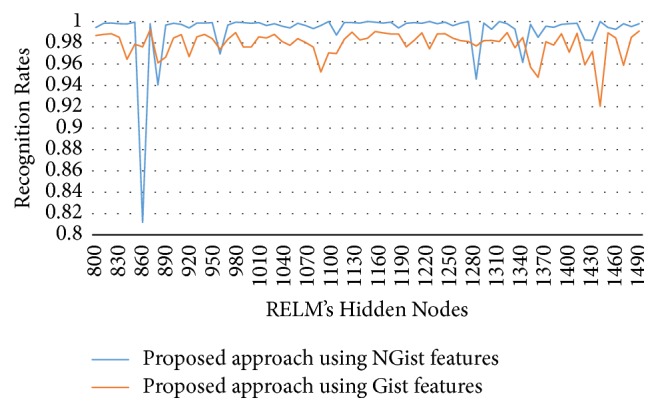
Recognition rates of green spectral band using extracted features of NGist and Gist at different numbers of RELM's hidden nodes.

**Figure 10:**
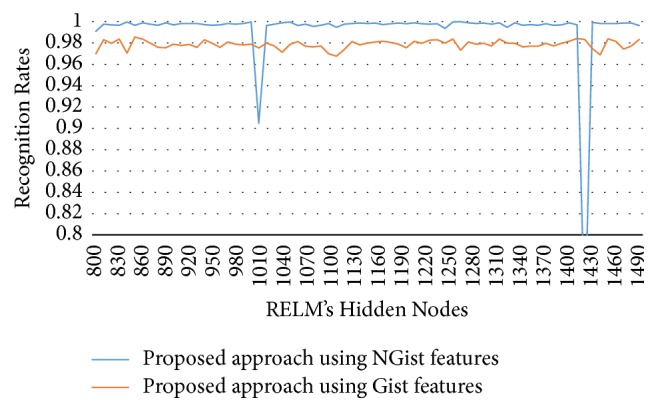
Recognition rates of red spectral band using extracted features of NGist and Gist at different numbers of RELM's hidden nodes.

**Figure 11:**
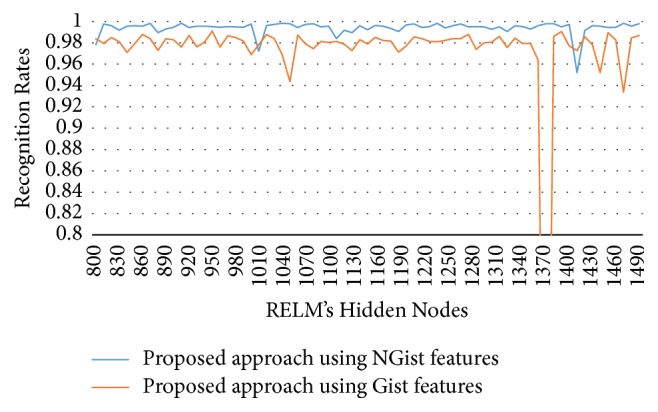
Recognition rates of NIR spectral band using extracted features of NGist and Gist at different numbers of RELM's hidden nodes.

To show the effectiveness of the proposed approach based on AE and RELM using NGist features over the features of the original Gist, we computed the recognition rates using extracted features of both descriptors at different numbers of RELM's hidden nodes. Figures 8–11 reveal the improvements of the proposed approach using NGist features over Gist features. For NGist features, we notice that the proposed approach achieves the maximum value of recognition rate (100%) or nearest to this value at different numbers of RELM's hidden nodes demonstrated in Figures 4–7. On the contrary, we note that the Gist features did not achieve the maximum value of recognition rate at any number of RELM's hidden nodes. The reason of this improvement is due to the ability of the proposed approach to cancel out the variations of extracted features computed from different blocks of different scales and orientations and improve the generalization to changes in palmprint images.

**Figure 12:**
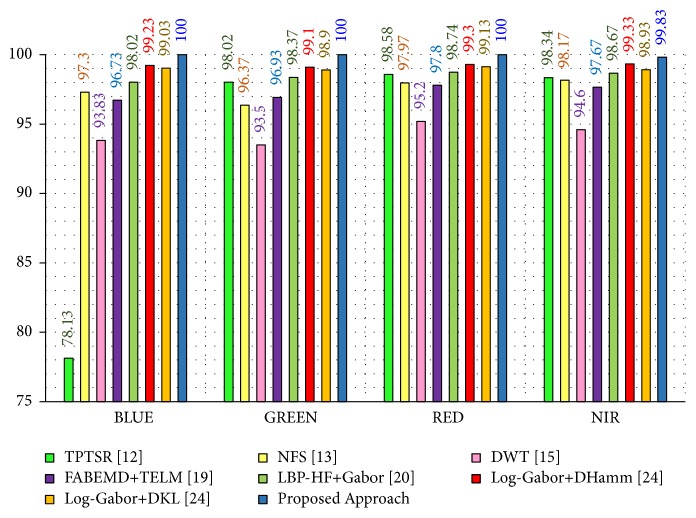
Recognition rates of experimental model I.

Moreover, we can see in Table 2 and Figure 12 that the proposed approach yields a highest recognition rate (100%) for the blue, green, and red spectral bands. Also, it achieves a recognition rate of 99.83% for the NIR spectral band. These results are highlighted in bold font in Table 2. Consequently, in the case of the blue spectral band, there is an improvement of 21.87% compared to TPTSR, 2.7% compared to NFS, 6.17% compared to DWT, 3.27% compared to FABEMD + TELM, 1.98% compared to LBP-HF + Gabor, 0.77% compared to Log-Gabor + *D*_Hamm_, and 0.97% compared to Log-Gabor + *D*_KL_.

In the case of the red spectral band, the results indicate that the proposed approach achieves also impressive improvements compared to the state-of-the-art approaches. Even though this experimental model mimics the real life situation where the system may be exposed to different conditions, the proposed approach attains an interesting result.

This is due to the strength of the proposed NGist descriptor for feature extraction. The approach has a high independency to changes in illumination and orientation problems. Moreover, the advantages of AE to deal with the nonlinearity of features and RELM to solve the overfitting problem made the power of the proposed approach.

For further evaluation of the proposed approach, the Equal Error Rate (EER) [5] is also used to assess the performance of the experimental results. It can be calculated according to the average of False Accepted Rates (FARs) and False Rejected Rates (FRRs) in different thresholds between 0 and 1. Usually, FAR is used to measure the possibility that the biometric approach accepts incorrectly an attempt by a user which is not registered as an authorized user. It is arithmetically computed as the ratio of the number of false acceptances divided by the number of recognition attempts. Contrarily, the FRR metric is used to measure the possibility that the biometric approach rejects incorrectly an attempt by a user who is indeed registered as an authorized user. FRR is computed as the ratio of the number of false rejections divided by the number of recognition attempts.  Table 3 exhibits the EERs of the proposed approach against the orientation based methods in the literature review, including competitive code, palm code, fusion code, ordinal code, and recent methods such as RLOC, BDOC–BHOG, HOC, and DOC on the four types of spectral bands. From Table 3, we can see that the proposed approach achieves the lowest EERs compared to the other methods in the state of the art on all spectral bands.

Additionally, we notice that the proposed approach achieves smaller EERs for the blue and green spectral bands than the red and NIR bands. The main possible reason is that the features of the palmprint in the red and NIR bands are very fine and need some level of sharpness to be more useful for recognition.

### 3.4. Experimental Model II

To demonstrate the robustness and efficiency of the proposed approach, we compare its results with some state-of-the-art approaches that follow this experimental model, namely, NFS [13], RBF [14], and LBP-HF + Gabor [20] on the same benchmark database. In this experimental model, the first three samples images of each palm from the first session are taken as a training dataset to form 1500 (500 × 3) images for each spectral band, and the other six samples images of each palm from the second session are used as a testing dataset of 3000 (500 × 6). This is done for red, green, blue, and NIR spectral bands, separately.

The results in Table 4 obviously demonstrate the advantage of the proposed approach in terms of effectiveness and robustness over other reported approaches. It offers attractive recognition rates of 99.70% to 100%, which are highlighted in a bold font in Table 4. With regard to the recognition rates of blue, green, red, and NIR spectral bands, it can be observed that the proposed approach yields improvements of 2.3%, 2%, 1.69%, and 1.13% compared to the LBP-HF + Gabor approach, which has the highest recognition rates against other approaches.

**Figure 13:**
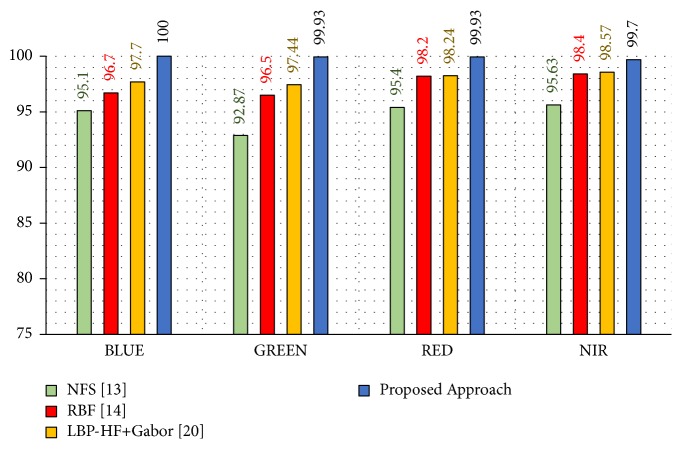
Recognition rates of experimental model II.

In addition to the recognition rates of our approach, the simplicity and efficiency compared to the LBP-HF + Gabor approach make it a robust and efficient approach. Furthermore, the small simple size of training dataset which are taken at different sessions and tested by a new test case is a rigorous challenge. Actually, the robustness of NGist descriptor with AE and RELM handles this challenge effectively.  Figure 13 visualizes the recognition rates of this work compared to the recent works that have high recognition rates.

### 3.5. Experimental Model III

Here, we take randomly three images of each different palm from the two sessions as a training dataset to form 1500 (500 × 3) images and the other nine images are taken as a testing dataset with 4500 (500 × 9) images. This model is done for each spectral band (blue, green, red, and NIR). The results are evaluated repeatedly for 30 times of random selection of the images in training and testing phases. The average recognition rates will be had as final results. The results of this model are compared with results of recent work given in [18].

**Figure 14:**
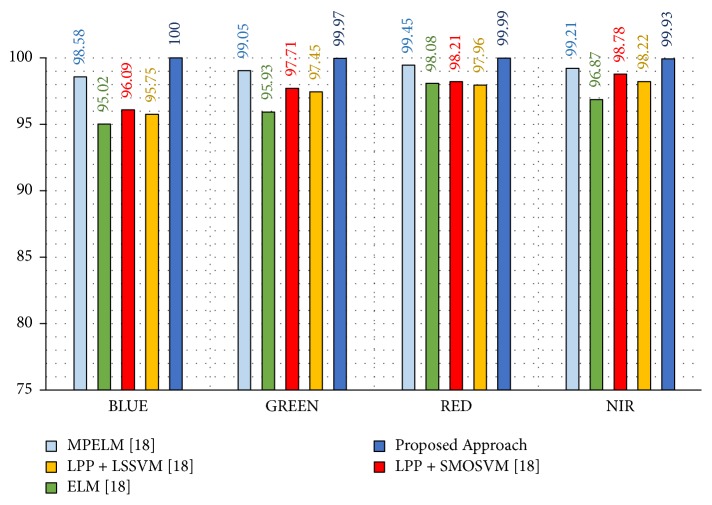
Recognition rates of experimental model III.

As we see in Table 5 and Figure 14, the proposed approach achieves high average recognition rates of 100%, 99.97%, 99.99%, and 99.93%, regarding blue, green, red, and NIR spectral bands, respectively. Similarly, it can be observed that the proposed approach yields improvements of 1.42%, 0.92%, 0.54%, and 0.72%, compared to the MPELM approach. These results indicate that the blue spectral band outperforms all other spectral bands effectively, whereas the red spectral band performs better than the green and NIR spectral bands.

### 3.6. Computational Cost

All experimental models have been implemented using MATLAB R2015a on a laptop with Windows 10 (x64), Intel Core i7-7500U with 2.7 GHz CPU processor, and 16 GB RAM. Execution time of feature extraction, feature reduction, and classification is shown in Table 6.

We notice that the average execution time of all steps is very small which makes the proposed approach is efficient and fast enough for real time condition.

## 4. Conclusion and Future Work

A novel multispectral palmprint recognition approach is proposed based on AE and RELM with an efficient extended version of Gist descriptor, named NGist. The NGist descriptor was applied to extract the features of palmprint, while AE was used to solve the problem of high dimensionality associated with the NGist features.

Recognition rate of the proposed approach has been evaluated using the public MS-PolyU database of multispectral palmprint images. The experiments were performed through three different experimental models, proving that the proposed approach attains higher recognition rates compared to the recent methods in the state of the art. Moreover, it has been observed that the blue spectral band outperforms all other bands effectively, whereas the red and green bands perform better than the NIR band in all three procedures.

Our next step is to extend the approach to handle spoofing problem by using multispectral palmprint fusion techniques. Additionally, we will investigate the applicability of pretrained deep learning models and transfer learning concept for biometric multispectral palmprint recognition.

## Figures and Tables

**Algorithm 1:**
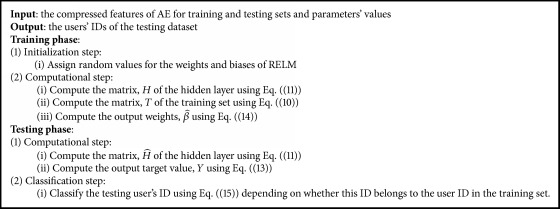
Multispectral palmprint classification based on RELM.

**Table 1:** Parameters setup.

Network Model	Parameters
AE	A number of hidden nodes of AE is *K*_AE_ = 250.
	Encoder and Decoder transfer function is a logistic sigmoid function.
	Maximum epochs = 10.
	L2WeightRegularization = 0.004.
	A loss function is a mean squared error function.
	Training algorithm is a conjugate gradient descent.

RELM	A number of hidden nodes of RELM, is
	*K*_RELM_ ∈ {800, 810, 820,…, 1500}.
	A regularization parameter (*λ*) = exp⁡(*val*), where
	*val* ∈ {−1, −0.9, −0.8,…, 0.9, 1}
	An activation function is a nonlinear sigmoid function, *g*(*x*) = (1/(1 + *e*^−*x*^))

**Table 2:** Comparison of recognition rates for the proposed approach with the state-of-the-art approaches for different spectral bands: blue, green, red, and NIR according to experimental model I.

Method [Ref.]	Recognition Rate (%)			
	Blue	Green	Red	NIR
TPTSR [12]	78.13	98.02	98.58	98.34
NFS [13]	97.30	96.37	97.97	98.17
DWT [15]	93.83	93.50	95.20	94.60
FABEMD + TELM [19]	96.73	96.93	97.80	97.67
LBP-HF + Gabor [20]	98.02	98.37	98.74	98.67
Log-Gabor + *D*_Hamm_ [24]	99.23	99.10	99.30	99.33
Log-Gabor + *D*_KL_ [24]	99.03	98.90	99.13	98.93
Proposed Approach	**100**	**100**	**100**	**99.83**

**Table 3:** The EERs (%) of the proposed approach against some orientation-based methods.

Method [Ref.]	ERRs (%)			
	Blue	Green	Red	NIR
Palm code [5]	0.0463	0.0507	0.0297	0.0332
Fusion code [21]	0.0212	0.0216	0.0179	0.0213
Ordinal code [22]	0.0202	0.0202	0.0161	0.0180
Competitive code [23]	0.0170	0.0168	0.0145	0.0137
RLOC [25]	0.0203	0.0249	0.0223	0.0208
BDOC–BHOG [26]	0.0487	0.0418	0.0160	0.0278
HOC [27]	0.0147	0.0144	0.0131	0.0139
DOC [28]	0.0146	0.0146	0.0119	0.0121
Proposed approach	**0.0001**	**0.0003**	**0.0007**	**0.0017**

**Table 4:** Comparison of recognition rates for the proposed approach with the state-of-the-art approaches for different spectral bands: blue, green, red, and NIR according to experimental model II.

Method [Ref.]	Recognition Rate (%)			
	Blue	Green	Red	NIR
NFS [13]	95.10	92.87	95.40	95.63
RBF [14]	96.70	96.50	98.20	98.40
LBP-HF + Gabor [20]	97.70	97.44	98.24	98.57
Proposed Approach	**100**	**99.93**	**99.93**	**99.70**

**Table 5:** Comparison of average recognition rates for the proposed approach with the state-of-the-art approaches for different spectral bands: blue, green, red, and NIR according to experimental model III.

Method [Ref.]	Average Recognition Rate (%)			
	Blue	Green	Red	NIR
MPELM [18]	98.58	99.05	99.45	99.21
ELM [18]	95.02	95.93	98.08	96.87
LPP + SMOSVM [18]	96.09	97.71	98.21	98.78
LPP + LSSVM [18]	95.75	97.45	97.96	98.22
Proposed Approach	**100**	**99.97**	**99.99**	**99.93**

**Table 6:** Average execution time of the proposed approach steps for one test sample (in seconds).

Step	Average execution time (s)
Feature extraction	0.237
Feature reduction	0.000024294
Classification	0.000317

## References

[1] Jain A. K. (2007). Technology: biometric recognition. *Nature*.

[2] Gomai A., El-Zaart A., Mathkour H. An efficient iris segmentation approach.

[3] Jaafar H., Ibrahim S., Ramli D. A. (2015). A robust and fast computation touchless palm print recognition system using LHEAT and the IFkNCN classifier. *Computational Intelligence and Neuroscience*.

[4] Binh Tran L., Le T. H. (2017). Multimodal Personal Verification Using Likelihood Ratio for the Match Score Fusion. *Computational Intelligence and Neuroscience*.

[5] Zhang D., Guo Z., Lu G., Zhang L., Zuo W. (2010). An online system of multispectral palmprint verification. *IEEE Transactions on Instrumentation and Measurement*.

[6] Han C., Cheng H., Lin C., Fan K. (2003). Personal authentication using palm-print features. *Pattern Recognition*.

[7] Wu X., Wang K., Zhang D. (2004). HMMs Based Palmprint Identification. *Biometric Authentication*.

[8] Raghavendra R., Dorizzi B., Rao A., Hemantha Kumar G. (2011). Designing efficient fusion schemes for multimodal biometric systems using face and palmprint. *Pattern Recognition*.

[9] Chen S. C., Fu H. G., Wang Y. (2012). Application of improved graph theory image segmentation algorithm in tongue image segmentation. *Computer Engineering and Applications*.

[10] Badrinath G. S., Kachhi N. K., Gupta P. (2011). Verification system robust to occlusion using low-order Zernike moments of palmprint sub-images. *Telecommunication Systems*.

[11] Gan J., Zhou D. A Novel Method for Palmprint Recognition Based on Wavelet Transform.

[12] Li J., Cao J., Lu K. (2013). Improve the two-phase test samples representation method for palmprint recognition. *Optik - International Journal for Light and Electron Optics*.

[13] Xu Y., Zhu Q., Fan Z., Qiu M., Chen Y., Liu H. (2013). Coarse to fine K nearest neighbor classifier. *Pattern Recognition Letters*.

[14] Zhang S., Gu X. (2013). Palmprint recognition based on the representation in the feature space. *Optik - International Journal for Light and Electron Optics*.

[15] Xu X., Guo Z., Song C., Li Y. (2012). Multispectral palmprint recognition using a quaternion matrix. *Sensors*.

[16] Lu G., Zhang D., Wang K. (2003). Palmprint recognition using eigenpalms features. *Pattern Recognition Letters*.

[17] Du F., Yu P., Li H., Zhu L. (2011). Palmprint recognition using gabor feature-based bidirectional 2dlda. *Communications in Computer and Information Science*.

[18] Xu X., Lu L., Zhang X., Lu H., Deng W. (2016). Multispectral palmprint recognition using multiclass projection extreme learning machine and digital shearlet transform. *Neural Computing and Applications*.

[19] Lu L., Zhang X., Xu X., Shang D. (2017). Multispectral image fusion for illumination-invariant palmprint recognition. *PLoS ONE*.

[20] El-Tarhouni W., Boubchir L., Al-Maadeed N., Elbendak M., Bouridane A. Multispectral palmprint recognition based on local binary pattern histogram fourier features and gabor filter.

[21] Kong A., Zhang D., Kamel M. (2006). Palmprint identification using feature-level fusion. *Pattern Recognition*.

[22] Sun Z. N., Tan T., Wang Y., Li S. Z. Ordinal palmprint represention for personal identification [represention read representation].

[23] Kong A. W.-K., Zhang D. Competitive coding scheme for palmprint verification.

[24] Bounneche M. D., Boubchir L., Bouridane A., Nekhoul B., Ali-Chérif A. (2016). Multi-spectral palmprint recognition based on oriented multiscale log-Gabor filters. *Neurocomputing*.

[25] Jia W., Huang D.-S., Zhang D. (2008). Palmprint verification based on robust line orientation code. *Pattern Recognition*.

[26] Hong D., Liu W., Su J., Pan Z., Wang G. (2015). A novel hierarchical approach for multispectral palmprint recognition. *Neurocomputing*.

[27] Fei L., Xu Y., Zhang D. (2016). Half-orientation extraction of palmprint features. *Pattern Recognition Letters*.

[28] Fei L., Xu Y., Tang W., Zhang D. (2016). Double-orientation code and nonlinear matching scheme for palmprint recognition. *Pattern Recognition*.

[29] Oliva A., Torralba A. (2001). Modeling the shape of the scene: a holistic representation of the spatial envelope. *International Journal of Computer Vision*.

[30] Siagian C., Itti L. (2008). Comparison of gist models in rapid scene categorization tasks. *Journal of Vision*.

[31] Li B., Cheng K., Yu Z. (2016). Histogram of oriented gradient based GIST feature for building recognition. *Computational Intelligence and Neuroscience*.

[32] Liou C.-Y., Huang J.-C., Yang W.-C. (2008). Modeling word perception using the Elman network. *Neurocomputing*.

[33] Liou C.-Y., Cheng W.-C., Liou J.-W., Liou D.-R. (2014). Autoencoder for words. *Neurocomputing*.

[34] Huang G. B., Zhu Q. Y., Siew C. K. Extreme learning machine: a new learning scheme of feedforward neural networks.

[35] Huang G. B., Zhu Q. Y., Siew C. K. (2006). Extreme learning machine: theory and applications. *Neurocomputing*.

[36] Xu J., Zhang W.-Q., Liu J., Xia S. (2015). Regularized minimum class variance extreme learning machine for language recognition. *EURASIP Journal on Audio, Speech, and Music Processing*.

